# Highly Bioavailable Forms of Curcumin and Promising Avenues for Curcumin-Based Research and Application: A Review

**DOI:** 10.3390/molecules25061397

**Published:** 2020-03-19

**Authors:** Sidney J. Stohs, Oliver Chen, Sidhartha D. Ray, Jin Ji, Luke R. Bucci, Harry G. Preuss

**Affiliations:** 1 School of Pharmacy and Health Professions, Creighton University Medical Center, Omaha, NE 68178, USA; 2 Biofortis Research, Addison, IL 60101, USA; oliver.chen@mxns.com; 3 Department of Pharmaceutical and Biomedical Sciences, Touro College of Pharmacy, Manhattan, NY 10027, USA; sidhartha.ray8@touro.edu; 4 PulchriBio Intl., Cambridge, MA 02138, USA; jji@pulchribio.com; 5 InnerPath Nutrition, Reno, NV 89523, USA; lukebucci@earthlink.net; 6 Department of Biochemistry, Georgetown University Medical Center, Washington, DC 220, USA; preusshg@georgetown.edu

**Keywords:** curcumin, curcumin metabolites, tetrahydrocurcumin, mechanisms of action, applications, solubility, absorption, bioavailability, hydrolysis, applications

## Abstract

Curcumin exerts a wide range of beneficial physiological and pharmacological activities, including antioxidant, anti-amyloid, anti-inflammatory, anti-microbial, anti-neoplastic, immune-modulating, metabolism regulating, anti-depressant, neuroprotective and tissue protective effects. However, its poor solubility and poor absorption in the free form in the gastrointestinal tract and its rapid biotransformation to inactive metabolites greatly limit its utility as a health-promoting agent and dietary supplement. Recent advances in micro- and nano-formulations of curcumin with greatly enhanced absorption resulting in desirable blood levels of the active forms of curcumin now make it possible to address a wide range of potential applications, including pain management, and as tissue protective. Using these forms of highly bioavailable curcumin now enable a broad spectrum of appropriate studies to be conducted. This review discusses the formulations designed to enhance bioavailability, metabolism of curcumin, relationships between solubility and particle size relative to bioavailability, human pharmacokinetic studies involving formulated curcumin products, the widely used but inappropriate practice of hydrolyzing plasma samples for quantification of blood curcumin, current applications of curcumin and its metabolites and promising directions for health maintenance and applications.

## 1. Introduction

Curcumin is the major biologically active polyphenolic constituent in turmeric, also called diferuloylmethane (1*E*,6*E*)-1,7-bis(4-hydroxy-3-methoxyphenyl)hepta-1,6-diene-3,5-dione) (https://pubchem.ncbi.nlm.nih.gov/compound/curcumin). Curcumin is found primarily in roots and rhizomes of the turmeric plant (*Curcuma longa*). In addition to curcumin, two other curcuminoids occur in lesser amounts in turmeric, namely, demethoxycurcumin (DMC) and bis-demethoxycurcumin (BDMC). Removal of the methoxyl groups yielding DMC and BDMC results in different chemical and biological properties as compared with curcumin. Curcuminoids make up 2-4% of dry turmeric root powder [1,2,3,4,5,6,7].

Curcumin can be administered as turmeric, concentrates of turmeric, essentially pure (95%) curcuminoids or curcumin alone. Unfortunately, all of these discrete, disparate entities have been called “curcumin”, causing much confusion in the literature. Curcumin exhibits a wide range of beneficial effects including anti-inflammatory, antioxidant, chemoprotective, tissue protective, antibacterial, anti-fungal, antiviral, metabolism regulating, immuno-modulating, antineoplastic and anti-depressant properties [1,2,3,4,5,6,7,8,9,10,11,12,13,14,15]. 

Issues which greatly limit the effectiveness and usefulness of curcumin are its low bioavailability attributed to water insolubility, and rapid metabolism to inactive metabolites. Curcumin is an oil-soluble compound, practically insoluble at room temperature in water at acidic and neutral pH. While it is soluble in alkali, it is very susceptible to auto-degradation. The water solubility of curcumin is estimated to be 11 ng/mL (https://pubchem.ncbi.nlm.nih.gov/compound/curcumin). Therefore, various formulations have been developed to enhance solubility or dispersibility with the goal of enhancing bioavailability and consequent bio-efficacy [14,15,16,17,18,19,20,21,22,23,24,25,26,27,28,29,30,31,32,33,34,35,36,37]. The reported delivery systems for curcumin include micelles, liposomes, phospholipid complexes, microemulsions, nano-emulsions, emulsions, solid lipid nanoparticles, nanostructured lipid carriers, biopolymer nanoparticles and microgels. They not only enhance efficacy [14,15,16,17,18,19,20,21,22,23,24,25,26,27,28,29,30,31,32,33,34,35,36,37,38,39,40,41,42], but also increase curcumin bioavailability by optimal permeation in the small intestine and preventing possible degradation in the gastrointestinal tract [43]. 

This review, as compared to other curcumin-related reviews, summarizes the three broad formulation strategies that have been employed to enhance absorption and bioavailability, providing specific examples for each category. It also reviews and contrasts the human pharmacokinetic studies that have been conducted with various formulations, and discusses the issues inherent in the majority of studies regarding the use of enzymatic hydrolysis of plasma samples prior to extraction and analysis. Finally, the potential health, wellness and therapeutic applications that can benefit from highly bioavailable curcumin are summarized. In this review, formulated curcumin products that provide >100-fold better absorption than unformulated curcumin are considered highly bioavailable. 

## 2. Formulation Strategies

The various formulations designed to enhance curcumin bioavailability can be divided broadly into a number of approaches with specific examples given. Curcumin is fat soluble, and therefore consuming curcumin with a fatty meal enhances absorption. Early approaches to enhancing absorption were the addition of turmeric oil (BCM-95^®^; BioCurcumax^®^; Curcugreen™) [17,18,19,20], a small amount of piperine (Curcumin C^3^ Complex^®^) [21,22,23] to stimulate the gastrointestinal system and prevent efflux of curcumin, or as a turmeric oleoresin (Curcugen™) which have resulted in small incremental increases in curcumin absorption.

Newer formulations have used adsorption and dispersion of curcumin onto various matrices as: γ-cyclodextrin (Cavacurmin^®^) [24]; whey-protein (CurcuminPro ^®^); rice flour, stearic acid, silica and magnesium stearate (CurcuFresh™); microcrystalline cellulose combined with soy lecithin phosphatidylcholine (Meriva^®^) [18,19,20,24,25,26]; spray-dried on porous silicon dioxide combined with triacetin and Panodan^®^ (Micronized Curcumin) [27]; cellulosic derivatives complexed with a hydrophobic carrier and natural antioxidants (CurcuWIN^®^) [20]; and a natural turmeric matrix formulation composed of carbohydrates, proteins, fiber and volatile oil (Acumin^®^/Cureit^®^) [19].

A number of formulations have utilized various techniques to decrease particle size as incorporation of curcumin in: liquid droplet nano-micelles containing lauroyl polyoxy 32-glycerides (Gelucire^®^) and polysorbate 20 (BioCurc^®^) [15]; fenugreek-derived galactomannan fiber (CurQfen^®^) [28]; liquid droplets containing Gelucire^®^ and caprylocaproyl polyoxyglycerides (Labrasol^®^), a BioCurc^®^ formulation [29]; colloidal dispersions using ghatti gum and glycerin (Theracurmin^®^) [30,31,32,33]; a matrix of glycerol esters of fatty acids, medium chain triglycerides, hydroxymethylcellulose and sodium alginate (MicroActive Curcumin®) [34]; a proprietary mixture of surfactants, polar lipids and solvents (HydroCurc^®^) [35]; solid lipid curcumin particles composed of docosahexaenoic acid (DHA), soy lecithin, stearic acid and vitamin C esters (Longvida^®^) [36]; a blend of sodium caseinate and Tween 20 [37]; proprietary microcapsules (Curcushine™); and a complex with acacia gum, quillaia (high in saponins) and sunflower oil (TurmiPure^®^). 

These formulation strategies are summarized in Table 1. In addition, the Table provides information on whether human pharmacokinetic studies have been published involving the various formulations, and whether enzymatic hydrolysis of plasma samples prior to extraction and analysis of curcumin were employed. Additional information regarding pharmacokinetic studies and enzymatic hydrolysis are provided below.

In addition to the above commercial formulations, a wide range of micellar and nano-particle formulations of curcumin have been prepared involving the use of ingredients as Tween 80, polysorbate 80, ceramic particles, polyethylene glycol (PEG), alginate, poly(lactic-co-glycolic acid) (PLGA), omega-3 fatty acids, chitosan and other substances [38,39,40,41,42]. Chemically modified variations of curcumin as well as conjugates in addition to the above formulations have been also developed. However, no pharmacokinetic studies have been reported to demonstrate the improvement in absorption and bioavailability [38,39,40,41,42]. As a consequence, only limited claims for greater bioavailability and efficacy can be justifiably made. Furthermore, it is not clear whether all of the ingredients used in these diverse formulations have generally recognized as safe (GRAS) status and can therefore be used in human subjects.

A number of the above listed formulations have no published human pharmacokinetic data but have claimed enhanced absorption and bioavailability on the basis of enhanced solubility in water or simulated gastric fluids. What these formulations fail to take into consideration is the solubility-permeability interplay in the gastrointestinal tract [44]. A solubility-enhancing formulation may result in a decrease in permeability, remain unchanged or even increase permeability. Therefore, a solubility-enhancing formula may not improve overall absorption [44]. Claims of enhanced bioavailability cannot be made solely on the basis of increased solubility. 

Delivery systems as micelles, liposomes, phospholipid complexes, microemulsions, nano-emulsions, emulsions, solid lipid nanoparticles, nanostructured lipid carriers, biopolymer nanoparticles and microgels exhibit greatest promise. They enhance efficacy [38,39,40,41,42], and also increase curcumin bioavailability by enhancing small intestine permeation, preventing possible degradation in the microenvironment, increasing plasma half-life and enhancing curcumin efficacy [43].

Cellular uptake of a substance depends on size and surface properties. A study compared the bioavailability of free curcumin powder and five curcumin formulations involving hydroxy propyl methyl cellulose, poly(lactic-co-glycolic acid) (PLGA), cyclodextrin, dendrimeric (globular, branched macromolecular structures) and magnetic [45]. The curcumin formulations had spherical particle sizes ranging from to ~ 5–58 nm, while unformulated curcumin exhibited highly aggregative, larger clusters (>1.2 μm). All formulations showed significantly higher cellular uptake comparing with free curcumin powder. The hydroxy propyl methyl cellulose-curcumin formulation, which had the smallest particle size of 5.2 nm and compared to other four formulations, displayed highest bioavailability and efficacy in prostate cancer cells. 

Surface properties of micro- and nano-particles such as charge and adhesion properties are also key factors for determining bio-absorption. Particles with neutral surface charge are rapidly recognized by the mononuclear phagocytic system as foreign entities and cleared out rapidly with a half-life of 3-5 min after intravenous administration [38,39,40,41,42]. Enhanced surface adhesion properties encourage contact with the intestinal mucosal epithelium which improves bioavailability. Thus, surface modifications are applied to generate surface charge or adhesion on the curcumin nanoparticles for improved bio-absorption. A slightly positive surface charge is preferred over a negative charge, since cell membranes are negatively charged, leading to interaction with a cell for internalization [38,39,40,41,42]. 

Various polymer or surfactant surface stabilizers such as polyvinyl alcohol (PVA), polyvinyl pyrrolidone (PVP) and surfactants sodium dodecyl sulfate (SDS), carboxymethylcellulose sodium salt, Tween 80, polysorbate 20 and D-α tocopheryl polyethylene glycol 1000 succinate (TPGS) have been utilized to achieve the stability of curcumin nanoparticles in physiological conditions. Their incorporation also increases permeability of cellular membranes which allows higher absorption of nanoparticles [38,39,40,41,42].

In addition to surface stabilizers, carriers with cellular affinity are conjugated to these nanoparticles to facilitate cellular uptake. Examples of carriers include but are not limited to poly lactic-co-glycolic acid (PLGA), Gelucire^®^, chitosan, glycerol monooleate (GMO), polycaprolactone (PCL), galactomannans, various cellulose derivatives and human serum albumin (HSA) [38,39,40,41,42]. Among these carriers, Gelucire^®^ in combination with polysorbate 20 appears to show the most promise toward clinical application due to its small particle size and high bioavailability [15].

## 3. Curcumin Chemistry and Metabolism

Growing knowledge of the complex metabolic fate of curcumin is at a point where much of the previous pharmacokinetics research is incomplete and outdated [46,47,48,49]. Most studies on curcumin that have observed exciting and strong biological effects have occurred as result of the administration of high levels of curcumin to cells in culture (in vitro) or to rodents. Unfortunately, these results provide marginal relevance to practical human application due to the fact that humans exhibit 4-16X greater phase II metabolism (conjugation with glucuronide and sulfate) than rodents [46].

Curcumin is a highly reactive compound because of its unique molecular structure. It is sensitive to visible and UV light, breaking down into a series of compounds with vanillin and ferulic acid being the most prominent end products [46,47,48]. Uncontrolled and unknown light exposure has cast a shadow of unreliability on curcumin research to date. Therefore, when studying curcumin, great care must be exercised to avoid exposure to light. 

Curcumin is unstable in aqueous solutions at pH > 6.5 at 37 °C through an autoxidation reaction, resulting in the formation of cleavage products with the ultimate products becoming vanillin and ferulic acid, cyclopentadione internal cyclization structures or dimerization compounds [46,47,48,49,50]. Curcumin can also be oxidized by free radicals and oxyradicals, with many decomposition products [46,47,48,49,50], accounting for some of the well-known antioxidant properties of curcumin. 

Once curcumin is ingested, several biological and chemical facts further decrease the delivery of curcumin to target sites. Curcumin is fat-soluble with no practical solubility in aqueous solutions more acidic than pH 6, an environment that enhances autooxidation [46,47]. Thus, human pharmacokinetic studies using unformulated curcumin unwittingly increase degradation through autoxidation before being absorbed. Nevertheless, epidemiological data on curry (turmeric)-eaters have shown significant health benefits from daily, low-dose, long-term ingestion of curcumin by traditional food practices [1,2,3,4,5,6,7,8,9]. Dietary practices developed over centuries involving heating turmeric in oil and then mixing with food in a meal may help its bioavailability.

In the gastrointestinal tract, curcumin binds tightly to mucus, further delaying epithelial cell uptake and subjecting curcumin to autooxidation and oxidative degradation [46]. Once transported into epithelial cells, extensive biotransformation occurs [46,50]. Phase I metabolism (mainly reduction) is the major transformation of curcuminoids in humans, forming dihydrocurcumin, tetrahydrocurcumin, hexahydrocurcumin and octahydrocurcumin [1,4,5,46,50]. Each of these forms as well as curcumin, DMC and BDMC are then subjected to Phase II conjugation with glucuronide, sulfate and glutathione [1,4,5,46,49]. Curcumin and its metabolites are subsequently effluxed into the intestinal lumen for elimination via defecation, the fate of most orally ingested curcumin [46]. 

Curcumin and its metabolites are subjected to further metabolism by the gut microbiome in the colon, resulting in multiple species with little chance of reabsorption [1,4,5,46,49]. The absorbed curcumin compounds are additionally metabolized by the hepatocytes, further converting them into reduced and conjugated forms for efflux into bile, with minor amounts entering circulation [46,49]. Once in circulation, curcumin and its metabolites adhere strongly to proteins, mostly albumin. 

**Figure 1:**
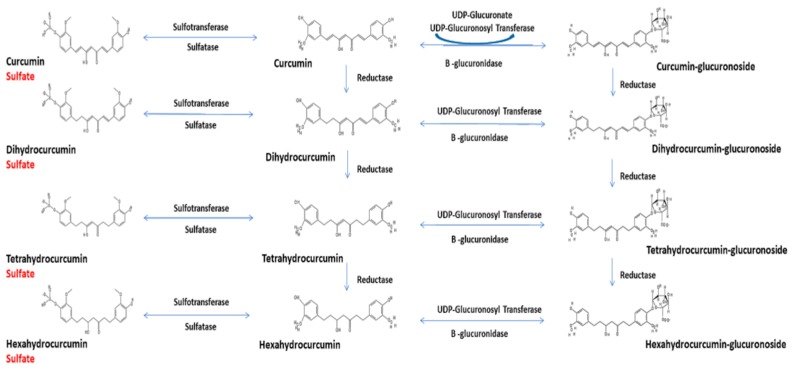
Metabolic pathways of curcumin.

Figure 1 shows the major known metabolites in human plasma after oral ingestion. While it illustrates curcumin metabolism, both DMC and BDMC also undergo the same metabolic pathways. Thus, ingestion of curcuminoids results in >42 total curcumin compounds in human and pure curcumin results in >13 metabolites. 

Pharmacokinetic studies in human have administered curcumin with and without DMC and BDMC. However, few human studies have assessed DMC and BDMC, even when they were quantified (total but not free) following enzymatic hydrolysis of the conjugated forms [24,25,26,27,35]. No studies have measured curcumin and all 13 metabolites that potentially occur after ingestion due to the absence of standards and the low quantities in the circulation.

## 4. Data Normalization and Inappropriate Hydrolysis of Plasma Samples

Curcumin is much more physiologically active than its conjugated and reduced metabolites, and is present in higher plasma levels in the free form than metabolites of its reduced forms. Therefore, the most accurate reflection of true bioavailability and bio-efficacy involves the determination of free curcumin [6,12,14,16]. The pharmacokinetic index used to determine extent (amount) of curcumin absorption is a plot of blood plasma concentration of the active constituent(s) against time, producing the area under the concentration-time curve (AUC). In addition, the C_MAX_ denotes the maximum concentration of curcumin in the plasma after dosing.

Pharmacokinetics data for curcumin micro- and nano-formulations are mostly available from preclinical studies. These limited data confirmed that curcumin formulations improved the bioavailability comparing to free, unencapsulated curcumin powder, as measured by the AUC or C_MAX_ of curcumin and its metabolites [38,39,40,41,42,46]. Due to large variations in administration, analytical techniques, dosage and other parameters, the AUCs and C_MAX_ data are not directly comparable across studies and formulations. However, when data from various pharmacokinetic studies are compared by normalizing the results based on AUC/mg and C_MAX_/mg of ingested curcumin, meaningful comparisons can be made [15,19,51,52], and clearly indicate that micro- and nano-formulations provide greatly enhanced bioavailability as compared to products that offer increased solubility [15,19]. 

A major issue is the fact that with few exceptions [15,19,36], most pharmacokinetic studies have hydrolyzed plasma samples prior to analysis, resulting in the determination of total and not just free curcumin [51,52]. Enzymatically hydrolyzing plasma with glucuronidase/sulfatase to free curcumin from its conjugates as opposed to measuring free curcumin in non-hydrolyzed plasma samples results in determining total curcumin and not free, bioactive curcumin. Thus, reporting total curcumin from hydrolyzed plasma samples provides greatly exaggerated and misleading results [51,52]. As a consequence, it is difficult to provide meaningful comparisons of curcumin absorption and bioavailability between products, unless direct comparative studies are conducted.

Various pharmacokinetic studies have compared absorption between a formulated product and unformulated curcumin [15,24,25,26,27,28,30,31,33] where enzymatic hydrolysis of plasma samples was employed, and therefore total curcumin but not free, bioactive curcumin was reported. Under these conditions, some indication of enhanced absorption is provided as a result of the product designed to enhance bioavailability. However, no information on plasma levels of free, bioactive curcumin or its bioactive reduction products is provided. A number of studies have also compared the pharmacokinetics of a curcumin formulation with earlier and more poorly absorbed formulations as curcumin with turmeric oil (BCM-95^®^; BioCurcumax^®^; Curcugreen™) or a small amount of piperine (Curcumin C^3^ Complex^®^), with contradictory results from original reports [18,19,20,24,25,26]. 

## 5. Health Promotion and Therapeutic Applications

A recent bibliometric study extensively reviewed the literature on the potential health benefits of curcumin, and found over 18,000 published regular manuscripts, reviews and meta-analyses, with half of them appearing within the last 5 years (after 2014) [1]. A high percentage of these studies were targeted towards various chronic diseases (e.g., cancers, diabetes, microbial, cardiovascular and neurological), and conditions associated with inflammation and oxidative stress [1]. The therapeutic potential of curcumin appears to provide overwhelming benefits compared to risks involved in the prevention and treatment of diseases. Curcumin may be beneficial and exhibit therapeutic actions in a broad range of conditions as: hepatotoxicity, cardiotoxicity, nephrotoxicity, pulmonary fibrosis, inflammatory bowel disease, ulcers, neoplastic conditions and multiple drug resistance [40]. Furthermore, curcumin exhibits wound healing, scar and cataract prevention, as well as metabolic regulation. In all these situations, poor oral absorption of curcumin has been a major complicating factor in assessing its full therapeutic potential. 

Curcumin administered with various drugs can be efficacious for treatment of many diseases and conditions [53]. Combination therapy reduced the signs and symptoms of diseases, and in many instances, prevented diseases. Curcumin, when administered in a wide variety of combinations and formulations, was shown to be active in almost all the vital organs in vivo, although its precise mechanism of action and bioavailability in various target tissues remains questionable. It is likely that when combined with other therapeutic treatments, curcumin can exert profound anti-inflammatory, antioxidative and antiproliferative properties. 

A noteworthy area where curcumin’s multipronged role is envisaged as quintessential is its ability to modulate a plethora of signaling pathways that operate in highly proliferating (cancer) cells. These comprise the nuclear factor kappa—B (NF-Κb), signal transducer and activator of transcription 3 (STAT3), Janus kinases/STAT (JAK/STAT), Wingless Int-1 (Wnt)/β-catenin, tumor necrosis factor (TNF), toll-like receptors (TLR), mitogen-activated protein kinase (MAPK), serine/threonine protein kinase (Akt, PKB), integrin and transforming growth factor-beta (TGF-β) pathways [54]. A surfeit number of carefully designed well-controlled studies have revealed that curcumin can inhibit various types of rapidly dividing tumor cells in culture, prevent carcinogen-induced cancers in rodent models and inhibit the growth of human tumors in xeno-transplant or ortho-transplant animal models. Based on these data, it is believed that the ability of curcumin to directly or indirectly modulate genome-dependent events (transcription and/or translation) makes it possible to exhibit anticancer or antiproliferative properties.

A large number of NIH-funded investigations are currently looking into other miscellaneous health benefits of curcumin, including its disease prevention abilities (https://projectreporter.nih.gov/reporter.cfm). These projects are at different stages (Phase I through Phase III) of execution. Curcumin also shows profound antitoxic properties in diverse target organs, such as, alleviation of neurotoxicity, cardiotoxicity, nephrotoxicity, hemato-toxicity, genotoxicity and hepatotoxicity [13,55]. Additional attributes that curcumin offers are its capacity to confer properties of electron receptors which destabilize reactive oxygen species (ROS), therefore explaining its antioxidant and anti-cell death (necrotic, apoptotic or aponecrotic) effects. Curcumin also boosts the sensitization of cancer cells to chemotherapy without exercising additional side effects, a provocative area for continued investigations [56].

A unique dimension of curcumin is its ability to influence a number of different target molecules such as adhesion molecules, pro-apoptotic and anti-apoptotic proteins, inflammatory factors, transcription factors, growth factors and a wide variety of enzymes and different kinases. Several elegant studies have demonstrated that curcumin can selectively influence pro- and anti-inflammatory factors such as NF-κB, STAT3, lipoxygenase, cyclooxygenase, xanthine oxidase and inducible nitric oxide synthase, resulting in sensitization of cancer cells to chemotherapeutic drugs and minimize the chemotherapy-induced side effects and toxicity [57,58,59]. In this regard, curcumin-mediated enhanced expression of pro-apoptotic molecules such as BAK and BID, and reduced expression of anti-apoptotic molecules such as BCL-2, BCL-XL and MCL-1 in the chemotherapy-treated cancer cells remains an active area of research. Furthermore, curcumin’s synergistic effect on docetaxel to activate tumor suppressor p53 and inhibit phosphoinositide 3-kinase (PI3K)/AKT, epidermal growth factor receptor (EGFR) and human epidermal growth factor receptor type 2 (HER2) are noteworthy reports [53,55,57].

With the development of curcumin formulations with very high bioavailability, it should now be possible to exploit these activities in Alzheimer’s disease, multiple sclerosis, Parkinson’s disease and amyotrophic lateral sclerosis (ALS) [38,39,40,41,42]. For example, curcumin formulations that were found to prevent accumulation of amyloid beta protein plaque and neurofibrillary tangles in animal models of Alzheimer’s disease are exceedingly promising [60,61]. Other neurological disorders that can benefit from highly bioavailable curcumin formulations include traumatic brain injury (TBI) [62] and neuropsychiatric disorders as post-traumatic stress disorder (PTSD), depressive disorders, psychotic disorders, bipolar disorder, obsessive-compulsive disorder (OCD) and autism [63]. 

A growing number of studies indicates that curcumin is beneficial in pain relief as osteoarthritis [64,65], spinal cord injury, diabetic neuropathy, sciatic nerve injury, chemotherapy induced peripheral neuro-inflammation [66] and migraine [67]. However, nearly all studies involving pain management have been conducted with unformulated curcumin or formulated products that offer some increased bioavailability but are not highly bioavailable. Thus, a tremendous opportunity exists for treating pain with highly bioavailable curcumin formulations.

An opioid crisis exists due to their over-use and highly addictive nature. Highly bioavailable curcumin may be an effective opioid replacement when appropriately dosed. An alternative approach to using curcumin as a stand-alone replacement may be the use of curcumin in combination with NSAIDS as naproxen, ibuprofen or acetaminophen, or in combination with cannabidiol (CBD). Furthermore, highly bioavailable forms of curcumin may be effective as replacements for NSAIDS, thus reducing the incidence of adverse effects as hepatotoxicity, nephrotoxicity and gastric ulcers.

## 6. Discussion

Based on available pharmacokinetic data, micellar and micronized formulations of curcumin appear to provide greatest absorption and bioavailability of free, bioactive curcumin and its reduced metabolites with >100-fold enhanced absorption as compared to unformulated curcumin. To date, the curcumin formulation comprised of liquid droplet nano-micelles containing Gelucire^®^ and polysorbate 20 (BioCurc^®^) has been shown to have the highest bioavailability with an absorption >400-fold as compared to unformulated curcumin [15]. Formulations that rely on increased aqueous solubility may enhance absorption of curcumin by a factor of approximately <10-fold, but the majority of these formulations have not been tested in humans.

Most pharmacokinetic studies with curcumin have used enzymatic hydrolysis of plasma samples prior to analysis, determining total and not just free bioactive curcumin in the plasma, and failing to employ the standard pharmaceutical model. Some data can be obtained from hydrolyzed plasma samples when formulated curcumin products are compared directly with unformulated curcumin. However, the plasma levels of free bioactive curcumin and its bioactive metabolites are not assessed and cannot be determined, and the data are misleading and greatly exaggerated.

Due to its profound antioxidant, anti-proliferative and anti-inflammatory properties, curcumin exerts a plethora of beneficial health effects. In broad general terms, curcumin exhibits tissue protective, analgesic and anti-neoplastic activities. Studies are also needed to determine the potential health-promoting effects in humans of the curcumin metabolites DMC, BDMC, tetrahydrocurcumin and hexahydrocurcumin.

The recent development of highly bioavailable forms of curcumin as BioCurc^®^ now permits its full therapeutic potential to be determined. Each delivery system has its pros and cons pertaining to specific applications. These delivery systems may ultimately point to an optimal regimen with the best dosage, maximum effectiveness and minimal or no side effects. It will remain a fertile area of research as the landscape of therapeutic and wellness potential of curcumin continues to unfold. 

## 7. Conclusions 

With the availability of highly bioavailable forms of curcumin that have >100-fold better absorption than unformulated curcumin, appropriate human pharmacological studies can now be conducted, knowing that desirable blood levels of the active forms of curcumin are achievable. Using appropriate analytical methods involving direct plasma extraction and devoid of enzymatic hydrolysis of plasma samples prior to extraction will enable accurate pharmacokinetic assessments of the bioactive forms of curcumin and determination of appropriate dosing relative to desired outcomes. The existence of highly bioavailable forms of curcumin now enables the exploration and determination of the full health-promoting benefits of curcumin.

## Figures and Tables

**Table 1:** Curcumin Formulation Strategies to Enhance Absorption.

Technical Approaches	Commercial Products	PK Studies	Hydrolysis	References
**A. Lipid Additions**				
Turmeric oil	BCM-95^®^; BioCurcumax^®^	Yes	Yes	17–20
Piperine	Curcumin C3 Comples^®^	Yes	Yes	21–23
Turmeric oleoresin	Curcugen^®^	No	NA	No
**B. Adsorption and Dispersion on Matrices**				
ɣ-Cyclodextrin	Cavacurmin^®^	Yes	Yes	24
Microcrystalline cellulose/Lecithin	Meriva^®^	Yes	Yes	18–20,24–26
Cellulosic derivatives	CurcuWIN^®^	Yes	Yes	20
Silicon dioxide/triacetin/Panodan®	Micronized Curcumin	No	NA	27
Carbohydrates/protein/oil/Fiber	Cureit^®^; Acumin^®^	Yes	No	19
Whey protein	CurcuminPro™	No	NA	No
Rice flour/ silica/magnesium/Stearate	Curcufresh™	No	NA	No
**C. Particle Size Reduction**				
Gelucire®/Polysorbate 20	BioCurc^®^	Yes	No	15
Galactomannan fiber	CurQfen^®^	Yes	yes	28
Gelucire®/Labrasol®	No	No	NA	29
Ghatti gum/glycerin/Lipids/hydroxymethyl	Theracurmin^®^	Yes	Yes	30–33
cellulose/sodium alginate	MicroActive Curcumin™	No	NA	34
Surfactants/polar lipids Solvents	HydroCurc™	No	NA	35
Docosahexaenoic acid/Lecithin/stearic acid	Longvida^®^	Yes	NO	36
Sod. Caseinate/Tween 80	No	No	NA	37
Proprietary microcapsules Curcushine™	Curcushine™	No	NA	No
Acacia gum/quillaia/sunflower oil	TurmiPure^®^	No	NA	No
